# Prognostic role of PHYH for overall survival (OS) in clear cell renal cell carcinoma (ccRCC)

**DOI:** 10.1186/s40001-021-00482-1

**Published:** 2021-01-19

**Authors:** Qiu Zhengqi, Guo Zezhi, Jiang Lei, Qiu He, Pan Jinyao, Ao Ying

**Affiliations:** Guangdong Key Laboratory of Genome Stability and Human Disease Prevention, Carson International Cancer Center, Department of Biochemistry and Molecular Biology, School of Basic Medical Sciences, Health Science Center, Shenzhen University, Shenzhen, 518060 China

**Keywords:** PHYH, Clear cell renal cell carcinoma, TCGA, Prognosis

## Abstract

This study attempts to evaluate the prognostic role of PHYH for overall survival (OS) in clear cell renal cell carcinoma (ccRCC) by means of publicly available data from The Cancer Genome Atlas (TCGA). Clinical pathologic features and PHYH expression were downloaded from the TCGA database and relationships between them were analyzed by univariate and multivariate Cox regression analyses. Gene Set Enrichment Analysis (GSEA) and gene–gene interactions were also performed between tissues with different PHYH expression levels. PHYH expression levels were significantly lower in patient with ccRCC compared with normal tissues (*p* = 1.156e−19). Kaplan–Meier survival analysis showed that high expression of PHYH had a better prognosis than low expression (*p* = 9e−05). Moreover, PHYH expression was also significantly associated with high grade (G2-4, *p* = 0.025), high stage (StageIII & IV, *p* = 5.604e−05), and high level of stage_T (T3-4, *p* = 4.373e−05). Univariate and multivariate Cox regression analyses indicated that PHYH could be acted as an independent prognostic factor (*p* < 0.05). Nomogram including clinical pathologic features and PHYH expression were also provided. GSEA revealed that butanoate metabolism, histidine metabolism, propanoate metabolism, pyruvate metabolism, tryptophan metabolism, PPAR signalling pathway, and renin–angiotensin system were differentially enriched in PHYH high-expression phenotype. ICGC database was utilized to verify the expression level and survival benefit of PHYH (both *p* < 0.05). We suspect that elevated PHYH expression may be served as a potential prognostic molecular marker of better survival in ccRCC. Besides, alpha-oxidation was closely regulated by PHYH, and PPAR signalling, pyruvate metabolism, butanoate metabolism, and RAS might be the key pathways regulated by PHYH in CCRC.

## Background

Clear cell renal cell carcinoma (ccRCC) is a major type of kidney cancer accounting for 90–95% of cases [1]. It sporadically arises from proximal tubular epithelial cells of the renal cortex, characterized by malignant epithelial cells with typical clear cytoplasm. During the past decade, data have shown a 2–3% yearly increase in ccRCC incidence. Recent advances in scientific medical research have led to an increased perception of the underlying pathophysiological molecular mechanism of ccRCC [2, 3]. The most common and vital characteristic associated with ccRCC and cancer in general is hypoxia. A condition that initiates a cascade of molecular events including angiogenesis and involves cell-cycle control proteins, which are closely associated with tumor growth [4, 5]. With regards to renal cell carcinoma (RCC), past researchers have identified that the hypoxia inducing factors 1α (HIF-1α) and its linked pathways such as ubiquitin–proteasome and rapamycin pathways are major contributors in RCC tumorigenesis [6–9]. More recent gene expression studies have identified some genes that predicts ccRCC aggressiveness and progression [10–13]. Yet, despite our efforts, no molecular biomarkers have been verified and potentially applicable in a clinical setting to move toward precision medicine of RCC treatment.

Phytanoyl-CoA 2-Hydroxylase gene (PHYH) is gene of the PHYH family and critical in the formation of peroxisomal protein which in turn assists in the alpha-oxidation of 3-methyl branched fatty acids. As immune system evasion is the hallmark of cancer, peroxisomes have an emerging role in the regulation of cellular immune response with reports showing pro-tumorigenic functions of peroxisome. However, there exists a significant gap in knowledge in the role of peroxisome and its associated gene PHYH in the potential of tumor induction and development [14].

Thus, the objective of the current study was aimed to evaluate the prognostic value of PHYH expression in human ccRCC data obtained from TCGA. Indeed, gene set enrichment analysis (GSEA) was performed to gain a better understanding into the underlying pathophysiological pathway mechanisms associated with ccRCC pathogenesis and its relationship with PHYH regulatory network. Potentially, discovering links and mechanisms connected to tumorigenesis.

## Methods

### RNA-sequencing patient data and bioinformatics analysis

High-throughput sequencing of gene expression data (HTSeq-counts) and clinical information of 538 cases of ccRCC and 72 para-cancerous cases were downloaded from TCGA official website (https://www.cancer.gov/about-nci/organization/ccg/research/structural-genomics/tcga). Normal ccRCC samples were excluded, and boxplots and whiskers plot were used to visualize expression differences for discrete variables [15].

### Gene set enrichment analysis

GSEA is bioinformatics method aimed to identify whether prior sets of genes or proteins are significantly different between two phenotypes [16]. Our study applied GSEA to generate an order list of all genes according to their correlation with PHYH expression, and significant survival differences observed between high and low PHYH groups were elucidated. Gene set permutations were performed 1000 times for each analysis. The expression level of PHYH was used as a phenotype label. The nominal *p *value and normalized enrichment score (NES) were used to sort the pathways enriched in each phenotype.

### Gene-network analysis

To investigate associated genes in performing different molecular function and biological pathway, gene interaction analysis was performed for the PHYH gene. Gene cards database (http://www.genecards.org) was used for searching gene–gene interaction network to identify gene–gene association, and then, we selected those that have a confidence value of 0.7 (high confidence) or higher. Furthermore, these set of genes were displayed using interactive gene view software (http://software.broadinstitute.org/software/igv).

### Nomogram model analysis

R (v3.4.3) was used to perform all statistical related analysis. Relationship between clinical pathological features and PHYH expression were analyzed via Wilcoxon signed-rank test and logistic regression. Nomogram construction was performed according to the guidelines proposed by Iasonos [17]. To identify independent prognostic predictors, we used a Cox proportional hazard regression model for univariable and multivariable analyses by the “Enter” method. The nomogram was developed to predict the 3 and 5 year prognosis mainly based on the results of the multivariable Cox regression model. The performance of the nomogram was estimated regarding discrimination and calibration. The C-index was applied to evaluate discrimination [18], which refers to the models’ ability to accurately distinguish the outcomes. A higher C-index indicates more precise model predictions [19]. Calibration curves were performed by comparing the means of the nomogram-predicted outcomes with the actual outcomes estimated with Kaplan–Meier. The bootstrapping (1000 repetitions) method was applied to reduce the estimate bias. In addition, model validations were performed using the data of the validation ccRCC cases as follows. First, we calculated the total points of the patients in the validation group using the established nomogram. Next, we used the total points as a factor to perform Cox regression analysis. Finally, the C-index and calibration curves were developed with the results of regression analysis. Receiver-operating characteristics (ROCs) curve was used for the sensitivity and specificity of nomogram.

### Statistical analysis

All statistical analyses were conducted using R (v.3.4.3). The relationship between clinical pathologic features and PHYH were analyzed with the Wilcoxon signed-rank test and logistic regression. Clinicopathologic characteristics associated with overall survival in TCGA patients using Cox regression and the Kaplan–Meier method. Multivariate Cox analysis was used to compare the influence of PHYH expression on survival along with other clinical characteristics (age, gender, race, grade, and Stage). The cut-off value of PHYH expression was determined by its median value.

## Results

### Association with PHYH expression and clinicopathologic variables

**Fig. 1:**
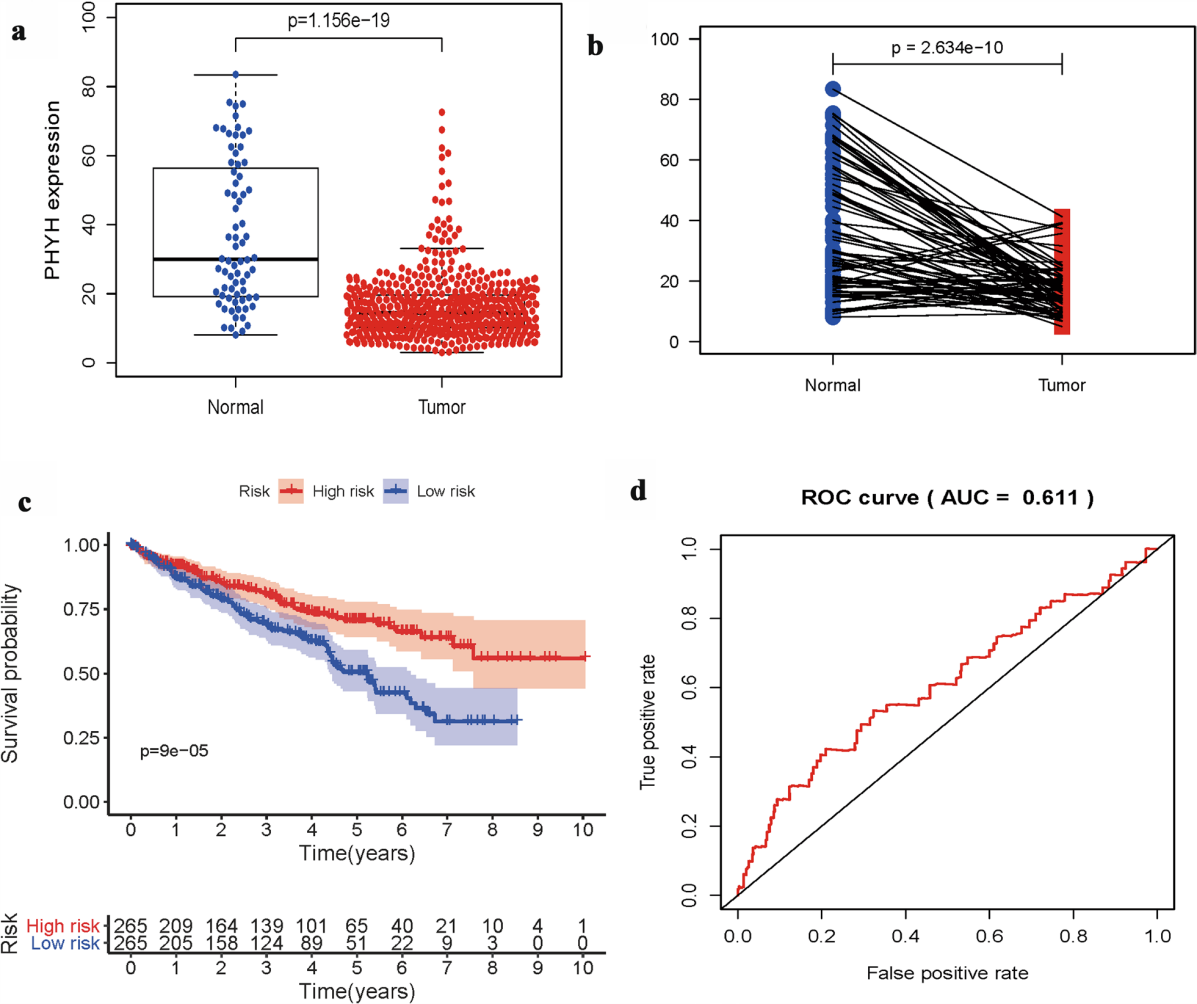
**a** Boxplot showed that the PHYH expression in ccRCC tissues (*n* = 538) was different from that in para-cancerous tissues (*n* = 72) in TCGA dataset; **b** pairwise boxplot showed that the PHYH expression in ccRCC tissues (*n* = 72) was also different from that in matched para-cancerous tissues (*n* = 72) in TCGA dataset; **c** Impact of PHYH expression on overall survival in ccRCC patients in TCGA cohort. **d** ROC curve analysis of significantly PHYH expression between normal patients and ccRCC patients with tumor

**Fig. 2:**
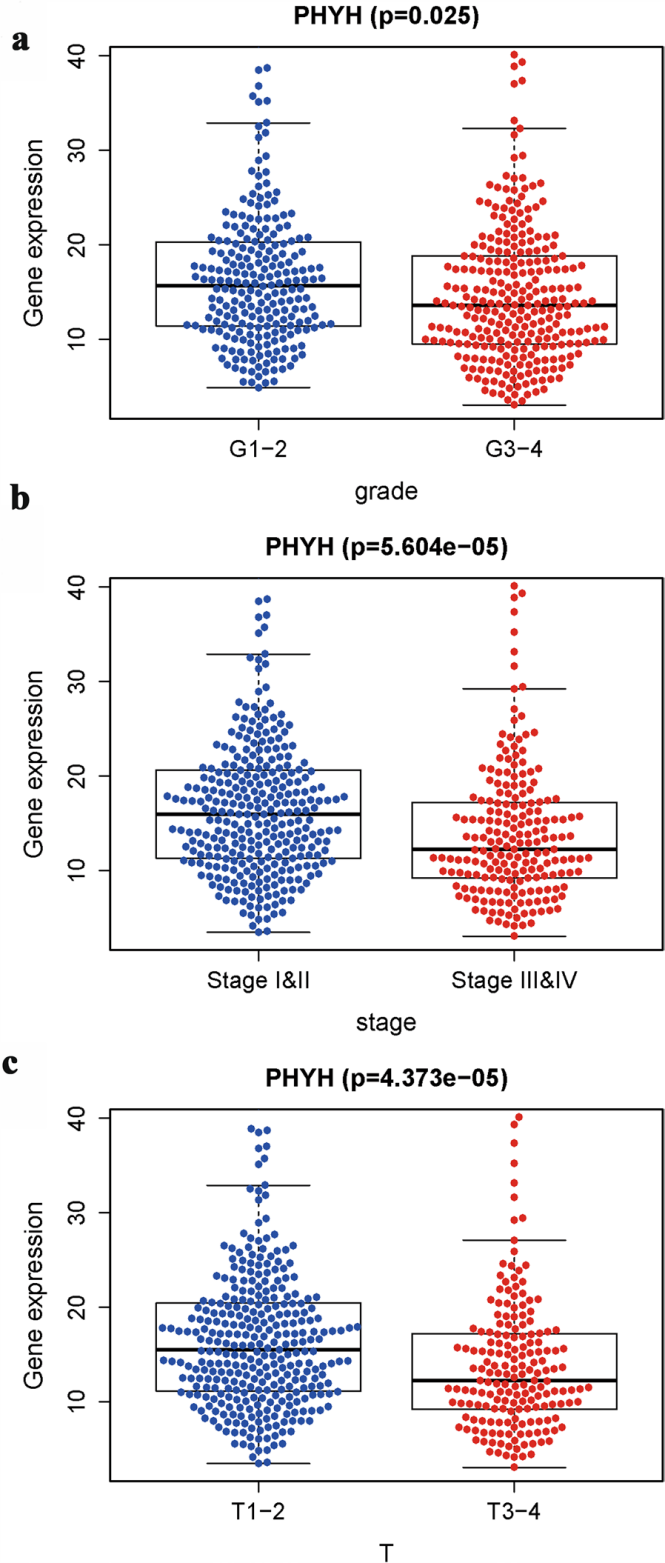
Association with PHYH expression and clinicopathologic characteristics, including **a** grade, **b** stage, and **c** primary tumor size (T)

As shown in Fig. 1, expression of PHYH is significantly lower in patients with tumor (*p* = 1.156e−19 & *p* = 2.634e−10). Classic univariate ROC curve analysis was performed to assess true-positive rate and false-positive rate of the PHYH expression between adjacent non-neoplastic kidney tissue and tumor based on the are under the curve (AUC). The results revealed that PHYH expression had a reasonable AUC of 0.611. In addition, decreased expression of PHYH correlated significantly with grade of cancer cells (G1-2 vs. G2-4, *p* = 0.025), the Union for International Cancer Control (UICC) stage (Stage I&II vs. Stage III&IV, *p* = 5.604e−05), and size of primary tumor (T1-2 vs. T3-4, *p* = 4.373e−05) (Fig. 2a–c, Table 1).

**Table 1:** PHYH expressions associated with clinical pathological characteristics (logistic regression)

Clinical characteristics	Total (N)	Odds ratio in PHYH expression	*p* value
Age (> 65 vs. ≤ 65)	511	1.347 (0.933–1.949)	0.112
Gender (Female vs. Male)	511	0.594 (0.410–0.856)	*0.005*
Race (African vs. White)	511	0.368 (0.069–1.670)	0.203
(Asian vs. White)		0.586 (0.317–1.060)	0.081
Grade (G3-4 vs. G1-2)	511	0.591 (0.416–0.840)	*0.003*
Stage (Stage I&II vs. Stage III&IV)	511	0.506 (0.352–0.725)	*0.000*
T (T1-2 vs. T3-4)	511	0.529 (0.365–0.762)	*0.001*
M (M0 vs. M1&X)	511	0.976 (0.635–1.501)	0.913
N (N1&X vs. NO)	511	0.740 (0.521–1.049)	0.091

The numbers marked in italics are less than 0.05, and the corresponding *p* value is statistically significant

### Survival outcomes and multivariate analysis

**Fig. 3:**
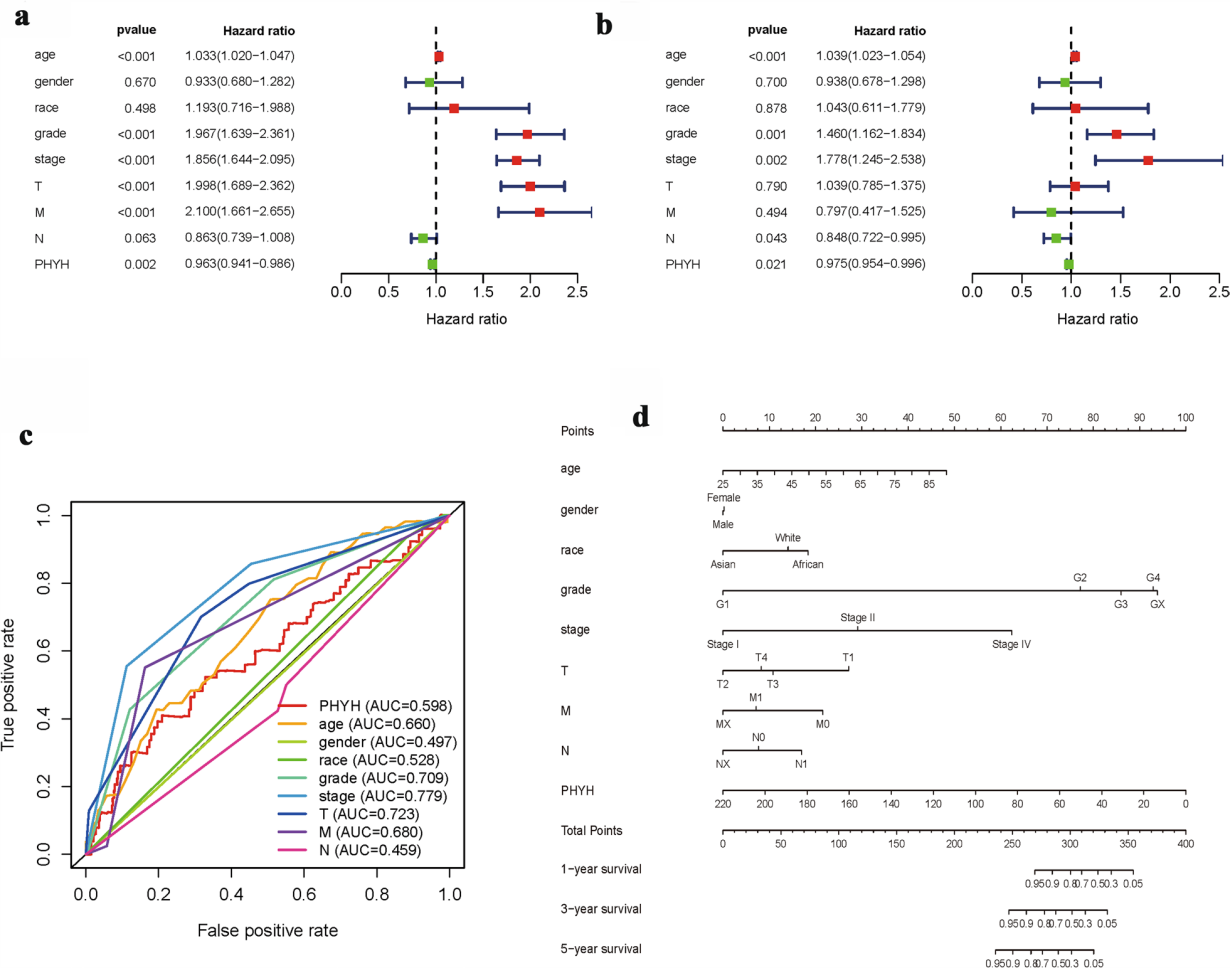
**a** Univariate Cox regression analysis of PHYH expression and clinicopathologic characteristics; **b** multivariate Cox regression analysis of PHYH expression and clinicopathologic characteristics; **c** ROC curves analysis of PHYH expression and clinicopathologic characteristics. **d** Nomogram of PHYH expression and clinicopathologic characteristics

The Kaplan–Meier survival analysis (Fig. 1c) showed that ccRCC with low expression of PHYH had a worse prognosis than that with high expression of PHYH ( p = 9e−5). The univariate analysis revealed that positive distant metastasis is correlated significantly with a poor overall survivability (hazard ratio [HR]: 2.1; 95% confidence interval [CI]: 1.661–2.655; *p* < 0.001). Other clinicopathologic variables associated with poor survival include age, grade, UICC stage, size of primary tumor, and PHYH expression (Fig. 3s, Table 2). At multivariate analysis, factors such as age, grade, stage, and PHYH expression remained associated with overall survival (Fig. 3b, Table 2). Classical univariate ROC curve analyses revealed that grade, stage, and size of primary tumor (T) showed a high AUC of 0.7, 0.779, and 0.723, respectively (Fig. 3c).

**Table 2:** Associations with overall survival and clinicopathologic characteristics in TCGA patients using univariate and multivariate Cox analyses

Clinical characteristics	univariate analysis		Multivariate analysis	
	HR (95% CI)	*p* value	HR (95% CI)	*p* value
Age	1.033 (1.020–1.047)	*0.000*	1.039 (1.023–1.054)	*0.000*
Gender	0.933 (0.680–1.282)	0.670	0.938 (0.678–1.298)	0.700
Race	1.193 (0.716–1.988)	0.498	1.043 (0.611–1.779)	0.877
Grade	1.967 (1.639–2.361)	*0.000*	1.460 (1.162–1.834)	*0.001*
Stage	1.856 (1.644–2.095)	*0.000*	1.778 (1.245–2.538)	*0.002*
T	1.998 (1.689–2.362)	*0.000*	1.039 (0.785–1.375)	0.790
M	2.100 (1.661–2.655)	*0.000*	0.797 (0.417–1.525)	0.494
N	0.862 (0.739–1.008)	0.063	0.848 (0.722–0.995)	*0.043*
PHYH	0.963 (0.941–0.986)	*0.002*	0.975 (0.954–0.996)	*0.021*

The numbers marked in italics are less than 0.05, and the corresponding *p* value is statistically significant

A point ranking system was also developed to rank the association of each factor with survivability (Fig. 3d). The higher the point for a give factor, the lower the survivability. As the results show, grade (G2, G3, and G4) and stage (I, II, and IV) are significantly associated with low survivability. In addition, higher age and lower PHYH expression are also significant related to low survivability. Interestingly, ethnic group African and white have lower survivability compared to Asian.

### Gene network

**Fig. 4:**
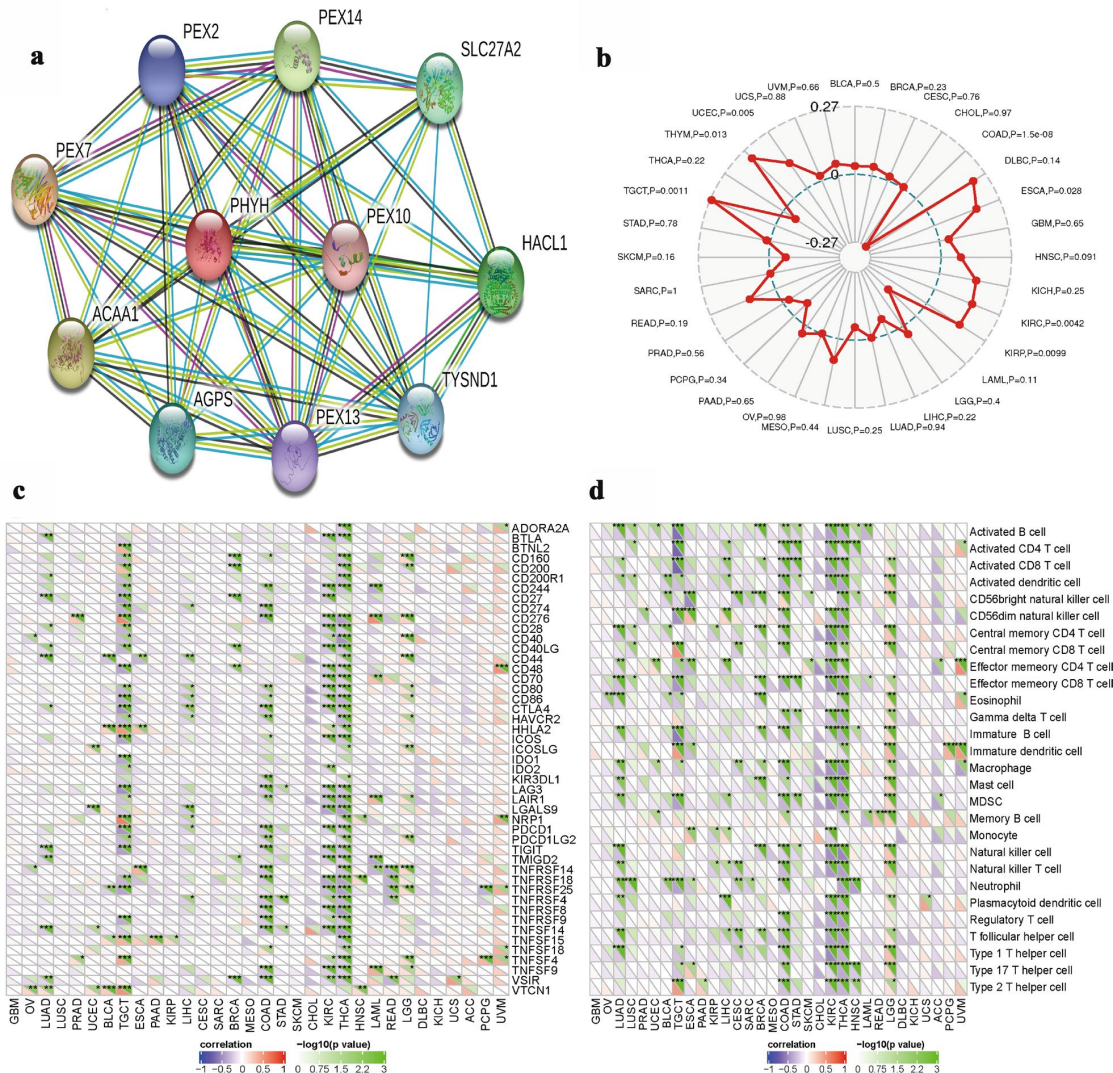
**a** PPI network of PHYH in ccRCC cases. **b** Relationships between PHYH and microsatellite instability (MSI). **c** Associations between PHYH and immune checkpoint inhibitors. **d** Relationships between PHYH and the methods of immunity

We also investigate gene network to identify their gene–gene interaction. Our results showed that PHYH in connected to 10 different genes in gene–gene interaction (Fig. 4a). Associations are meant to be specific and meaningful; this does not necessarily mean that they are physically binding each other (Additional file 1: Fig.S1). Among these genes, 5 are PEX genes that encode peroxin proteins (PEX2, PEX7, PEX10, PEX13, and PEX14) which suggest the existence of protein interactions with PHYH in ccRCC. Figure 4b shows the relationships between PHYH and microsatellite instability (MSI). The associations between PHYH and immune checkpoint inhibitors are also displayed in Fig. 4c. Figure 4d presents the relationships between PHYH and the methods of immunity.

### GSEA identifies a PHYH-related signalling pathway

**Fig. 5:**
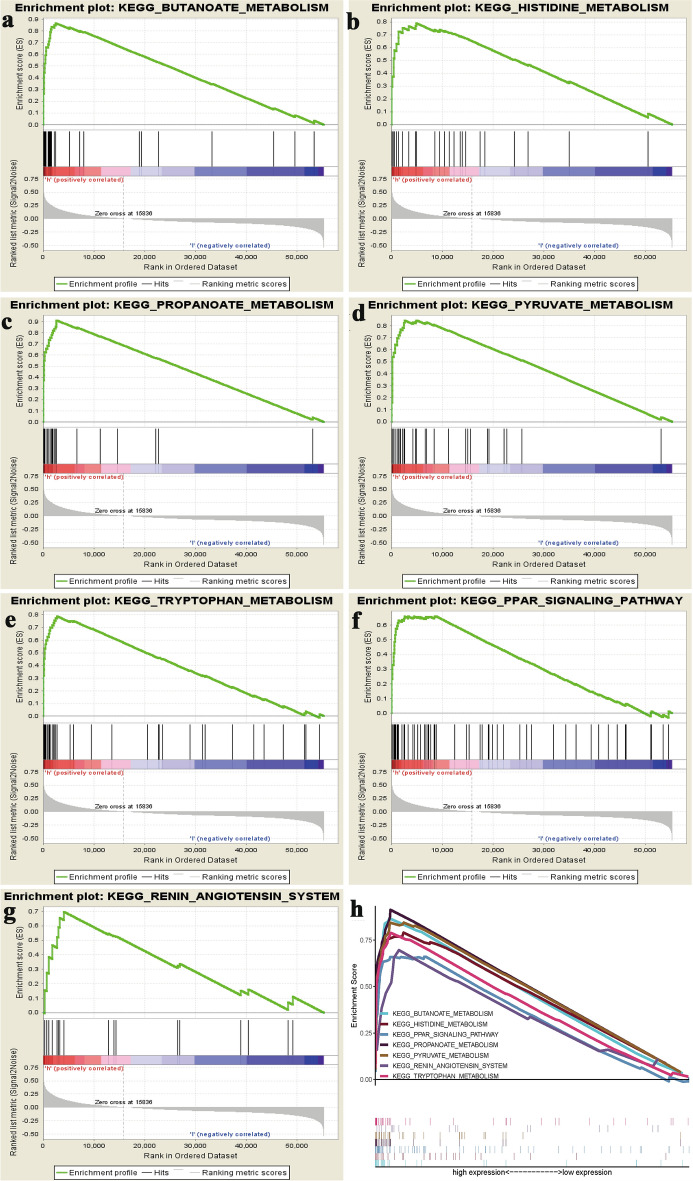
Enrichment plots from gene set enrichment analysis (GSEA). GSEA results showing **a** butanoate metabolism, **b** histidine metabolism, **c** propanoate metabolism, **d** pyruvate metabolism, **e** tryptophan metabolism, **f** PPAR signalling pathway, and **g** renin–angiotensin system are differentially enriched in PHYH-related ccRCC. **h** Comparison of enrichment plots for all significant pathways

To identify signalling pathways that are differentially activated in ccRCC, we conducted Gene Set Enrichment Analysis (GSEA) between low and high TFAP2B expression data sets. GSEA reveal significant differences (FDR b 0.05, NOM p-val b 0.05) in enrichment of MSigDB Collection (c2.cp.biocarta and h.all. v6.1. symbols). We selected the most significantly enriched signalling pathways based on their normalized enrichment score (NES) (Fig. 5, Table 3). Figure 5 shows butanoate metabolism, histidine metabolism, propanoate metabolism, pyruvate metabolism, tryptophan metabolism, PPAR signalling pathway, and renin–angiotensin system are differentially enriched in high PHYH expression phenotype.

**Table 3:** Gene sets enriched in phenotype high

MSigDB collection	Gene set name	NES	NOM p-val	FDR q-val
c2.cp.biocarta.v6.1.symbols.gmt	BUTANOATE_METABOLISM	2.506	0.000	0.000
h.all.v6.1.symbols.gmt	HISTIDINE_METABOLISM	2.503	0.000	0.000
	PPAR_SIGNALING_PATHWAY	2.124	0.000	0.006
	PROPANOATE_METABOLISM	2.500	0.000	0.000
	PYRUVATE_METABOLISM	2.551	0.000	0.000
	RENIN_ANGIOTENSIN_SYSTEM	2.071	0.002	0.008
	TRYPTOPHAN_METABOLISM	2.671	0.000	0.000

### Verification of PHYH in ccRCC

**Fig. 6:**
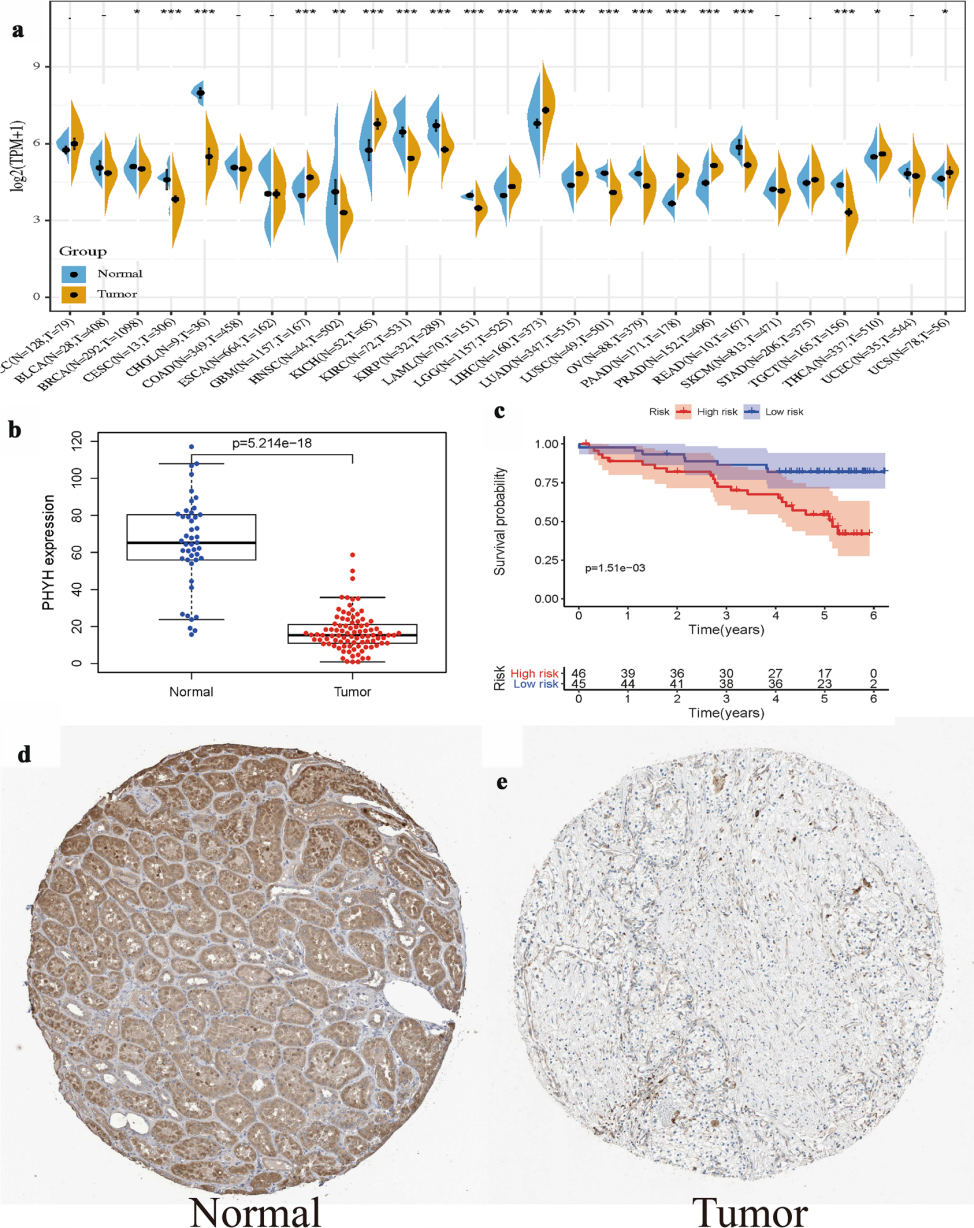
Verification of PHYH in ccRCC: **a** the expression level of PHYH in various cancers by GTEx and TCHA; **b** the boxplot of PHYH in ICGC database; **c** the survival analysis of PHYH in ICGC database; **d** the HPA database indicated the difference of immunohistochemistry in normal and kidney cancers

To further verify the expression level and survival benefit of PHYH in ccRCC, the GTEx, ICGC, and HPA databases were utilized, respectively. As displayed in Fig. 6a, the expression levels of PHYH in various cancers were shown including ccRCC with *p* < 0.001. In terms of ICGC database, the boxplot and survival analysis were consistent with in TCGA (*p* = 5.214e−18, *p* = 1.51e−03, respectively, Fig. 6b, c). The HPA database indicated the difference of immunohistochemistry in normal and kidney cancers (Fig. 6d, e).

## Discussion

The expression of PHYH has been linked to multiple diseases such as Refsum Disease and Retinitis Pigmentosa [20]. Although there are no study associating human cancers to PHYH expression, The Human Protein Atlas have reported it to be a prognostic marker in renal cancer [21]. To our knowledge, expression of PHYH and its impact on ccRCC has not yet been explored. Therefore, the potential role of PHYH in ccRCC was the main focus point of our study.

We applied bioinformatics analysis using high-throughput RNA-sequencing data from TCGA to examine PHYH expression in ccRCC patients and its association with various advanced pathologic characteristics. We demonstrated that a decrease PHYH expression is associated with presence of tumor, grade of cancer, stage of cancer, primary size of tumor, age, and presence of distant metastasis. To further investigate the functions of PHYH in ccRCC, we performed GSEA and gene–gene network using TCGA data. GSEA showed that butanoate metabolism, histidine metabolism, propanoate metabolism, pyruvate metabolism, tryptophan metabolism, PPAR signalling pathway, and renin–angiotensin system are differentially enriched in PHYH low-expression phenotype. Gene-gen network analysis revealed association of PHYH with multiple PEX genes. These evidences highlighted the potential of PHYH serving as a prognostic marker of prognosis and therapeutic target in ccRCC.

Results from our study showed a decreased expression of PHYH gene in patients diagnosed with ccRCC. The PHYH gene encodes the enzyme phytanoyl-CoA hydroxylase, which is required for the alpha-oxidation of branched chain [22, 23] and long-chain [24] fatty acids such as phytanic acid in peroxisomes [25]. Researchers suspect that phytanoyl-CoA hydroxylase potentially participates in determining the number of peroxisomes within cells and is involved in regulating their activities [26]. Peroxisomes are membrane bound organelle within the cytoplasm that is conserved across eukaryotic cells [27], and plays a vital role in peroxisomal fatty acid beta-oxidation metabolism and ROS (reactive oxygen species) conversion [28]. Diseases such as the Zellweger syndrome and other genetic diseases occurs due to implications in the fatty acid beta-oxidation [29, 30]. Study have found that many chemicals designated as peroxisome proliferators can induce peroxisome proliferation, resulting in increase in fatty acid oxidation in liver cells which leads to tumors’ growth in rodents [31–33]. A study has observed an absence of peroxisome in epithelial cells of proximal tubule in cancer cells of renal cell carcinoma [34]. AS phytanoyl-CoA hydroxylase is coded by the PHYH gene and key component in peroxisome regulation, results of the present study agree with the provided evidence and suggest that decreased expression of PHYH gene is associated with the absence of peroxisomes in ccRCC patients.

The gene–gene interactions form the results of our study have shown associations of multiple PEX genes (PEX2, PEX7, PEX10, PEX13, and PEX 14) with PHYH. *PEX* genes encode peroxins, a class machinery protein required for proper peroxisome assembly [35]. Autosomal recessive loss of function mutations in the *PEX* genes can result in peroxisome biogenesis disorders in the brain bone kidney and liver [36–39]. Overexpression of PEX genes such as PEX2 can result in accumulation of ubiquitinated PEX5 which can promote pexophagy (autophagosomal degradation of peroxisomes) [14]. Decreased PEX5 levels are associated with both the onset of cancer in vivo [40], and sensitivity to exogenous H_2_O_2_ addition in hepatocarcinoma model systems in vitro [41]. Identification of PEX14-containing vesicles has connected peroxisomes biogenesis to mitochondrial mediation [42]. PEX7 facilitate matrix protein import, which significantly contributes to peroxisome membrane growth [43]. Notably, PEX7 has primarily been documented to directly shuttle PHYH to the peroxisomal matrix [25]. Given the importance of peroxisomal matrix protein import in normal cells, it could be anticipated that the expression and/or function of peroxisome matrix proteins might become aberrant in tumor cells [14]. Combining the results from our analysis with the evidence presented, a clear association can be observed in which PHYH expression affects the expression of PEX genes. This in turn causes perturbations in peroxisomes biogenesis, function, and structure.

The abnormal expression of PHYH in our tumor cells activates the immune checkpoint. When the immune checkpoint is activated, the Antigen cannot be presented to T cells, blocking the presentation of Antigen in the Tumor Immune Ring, thus inhibiting the immune function of T cells, which allows the tumor cells to escape immune surveillance and survive. Through the change of immune checkpoint, we can infer the specific changes of immune pathway, which can be used to judge the therapeutic effect of targeted drugs in the future.

Results from the network analysis also revealed that alteration of PHYH expression in ccRCC phenotype implicates the alpha-oxidation pathway. Genes HACL1 and SLC27A2 (shown to be associated with PHYH) are genes that code for protein 2-hydroxyacyl-CoA lyase 1 and very long-chain acyl-CoA synthetase, and both enzymes along with PHYH are critical enzymes in converting phytanic acid to pristanic acid. Recent review has highlighted that peroxisomal disorders affect phytanic acid and alpha-oxidation [44]. As most metabolism of phytanic acid occurs in the liver and kidney via alpha-oxidation, an alteration in PHYH expression will mostly likely implicate peroxisomal and subsequent alpha-oxidation. The highlights the alpha-oxidation as a target pathway for furfure studies in ccRCC.

GESA pathway analyses of TCGA data reveal multiple differentially expressed pathways in PHYH low-expression phenotype. Among these altered pathways, the key peroxisome proliferator-activated receptor gamma (PPARγ) pathway has been shown to be functionally expressed [45] in ccRCC and that increased PPARγ abundance correlates with reduced patient survival [46]. Gluconeogenesis associated pathways pyruvate and butanoate metabolism have also been shown to be downregulated in kidney cancer [47]. The renin–angiotensin system (RAS) was also demonstrated to be underexpressed in ccRCC. RAS is a hormone system known to maintain blood pressure and body fluids [48]. Recent literature has implicated a crucial role of the RAS in the development and maintenance of cancer, particularly its effects on cancer stem cells [49–52]. In addition, RAS deregulation was demonstrated as a renal cancer risk factor [53]. Collectively, evidences suggest that these altered pathways and metabolism are good association factors with ccRCC and starting points for understating in depth underlying pathophysiological mechanism of ccRCC phenotype.

In conclusion, PHYH expression may be a potential prognostic molecular marker of poor survival in ccRCC. Low PHYH expression in ccRCC patients is closely related with dysfunction, degradation, and absence of peroxides. This occurs alters alpha-oxidation pathway which may potentially be a targeted pathway for future studies, Moreover, PPAR signalling, pyruvate metabolism, butanoate metabolism, and RAS may be the key pathway regulated by PHYH in ccRCC. Further experimental validation should be performed to prove the biologic impact of PHYH.

## Conclusion

Elevated PHYH expression could be served as a potential prognostic molecular marker of better survival in ccRCC. Besides, alpha-oxidation was closely regulated by PHYH, and PPAR signalling, pyruvate metabolism, butanoate metabolism, and RAS might be the key pathways regulated by PHYH in CCRC.

## Supplementary Information

**Additional file 1: Fig. S1.** Edges represent protein–protein associations

## Data Availability

The datasets used and/or analyzed during the current study are available from the corresponding author on reasonable request.

## Abbreviations

OS: Overall survival
ccRCC: Clear cell renal cell carcinoma
TCGA: The cancer genome atlas
GSEA: Gene set enrichment analysis
PHYH: Phytanoyl-CoA 2-hydroxylase
PPAR: Peroxisome proliferator-activated receptor
ICGC: International cancer genome consortium
RCC: Renal cell carcinoma
HIF-1α: Hypoxia inducing factors 1α
NES: Normalized enrichment score
ROCs: Receiver-operating characteristics
AUC: Are under the curve
UICC: Union for International Cancer Control
MSI: Microsatellite instability
GTEx: The genotype-tissue expression
HPA: Human protein atlas
RAS: Renin–angiotensin system
PPARγ: Peroxisome proliferator-activated receptor gamma
