# Supplementary figures and images for: Prognostic role of PHYH for overall survival (OS) in clear cell renal cell carcinoma (ccRCC)
Source: Eur J Med Res. 2021 Jan 19;26:9. doi: 10.1186/s40001-021-00482-1 (PMC7816304; doi:10.1186/s40001-021-00482-1)

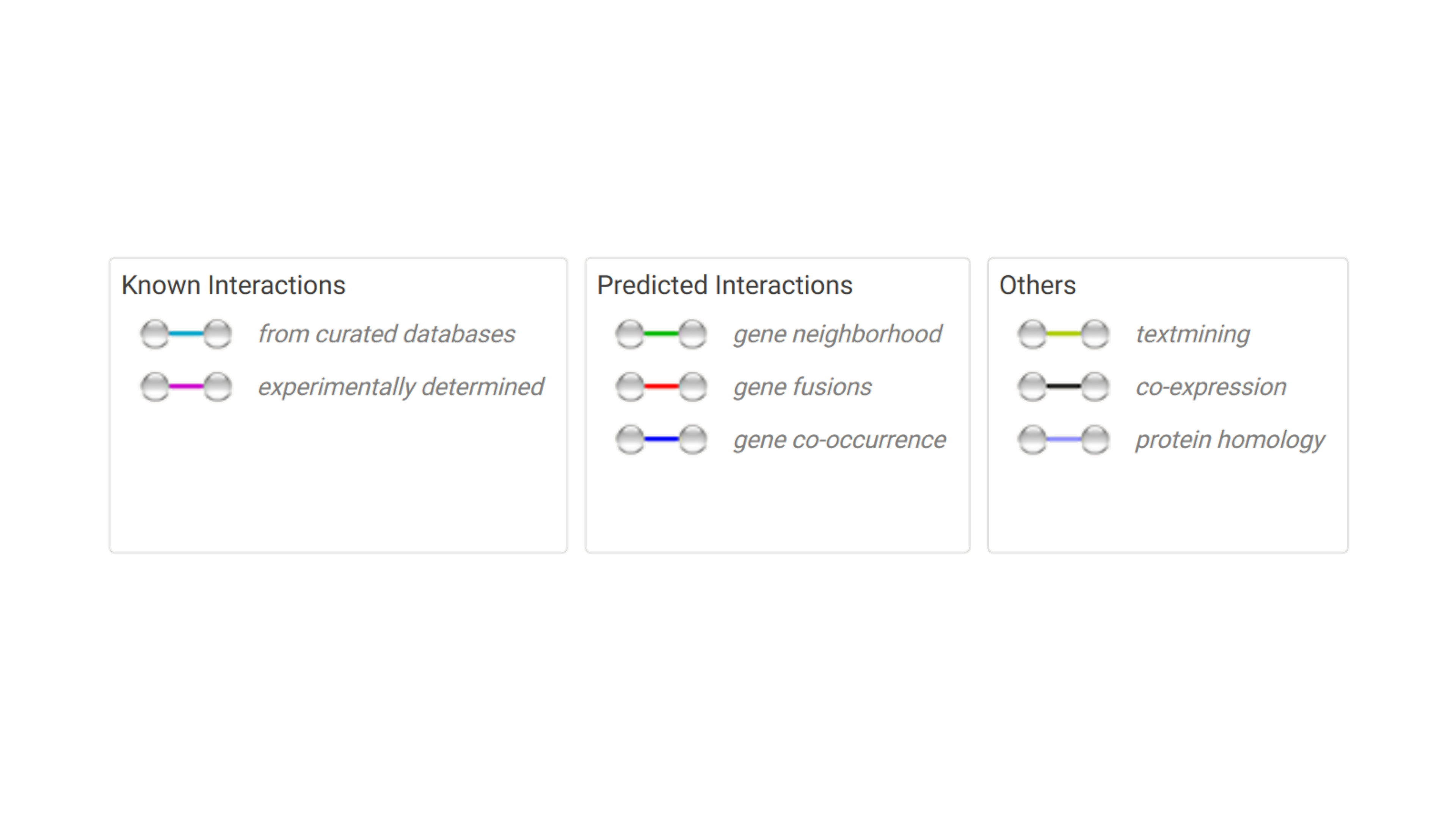

Supplement: Supplementary file 1 — Additional file 1: Fig. S1. Edges represent protein–protein associations [file 40001_2021_482_MOESM1_ESM.tif]
